# Reply: How well can frailty predict no benefit from ICD therapy?

**DOI:** 10.1002/clc.24230

**Published:** 2024-01-29

**Authors:** Michael Gotzmann, Marie Lewenhardt, Fabienne Kreimer

**Affiliations:** ^1^ Department of Cardiology and Rhythmology, St. Josef‐Hospital Ruhr University Bochum Bochum Germany

We would like to thank Zakiev et al.^1^ for their interest in our study. Zakiev et al.^1^ emphasized the importance of frailty in elderly patients when considering whether ICD implantation might have a benefit. We agree with their comment on key points.

A meta‐analysis on this topic was published a few years ago by Chen et al. Despite the lack of a clear definition of frailty, the meta‐analysis questioned the benefit of primary prophylactic ICD therapy in elderly frail patients.^2^ More recently, Segar et al. published a subgroup analysis of the SCD‐HeFT (Sudden Cardiac Death in Heart Failure Trial) from 2005, which demonstrated no benefit of ICD therapy in frailer patients.^3^

Overall, therefore, there is clear evidence of the importance of frailty in the care of older patients with an ICD. Nevertheless, there are no corresponding recommendations in the guidelines of the European Society of Cardiology. But why has too little attention been paid to the frailty of our patients when deciding whether ICD therapy is appropriate? In our opinion, this is essentially due to the lack of a universal definition of “frailty.”^4^ As our populations continue to age and frailty increases, such a clear definition is urgently needed.

Our study wanted to point out that there are large groups of patients for whom ICD therapy has no benefit.^5^ Unfortunately, it is difficult to analyze frailty in our study due to its retrospective character.

On more detailed analysis, it is certainly possible to better define the group of patients without potential benefit from ICD therapy. The consideration of frailty could provide an essential factor here.

## CONFLICT OF INTEREST STATEMENT

The authors declare no conflict of interest.
